# Effects of Six Processing Parameters on the Size of PCL Fibers Prepared by Melt Electrospinning Writing

**DOI:** 10.3390/mi14071437

**Published:** 2023-07-18

**Authors:** Yu Xie, Qi Fang, Han Zhao, Yang Li, Zhihai Lin, Jianxiong Chen

**Affiliations:** School of Mechanical Engineering and Automation, Fuzhou University, Fuzhou 350100, China; xieyu_me@fzu.edu.cn (Y.X.);

**Keywords:** melt electrospinning writing, polymer, orthogonal design, 3D printing

## Abstract

Melt electrospinning writing is a new and promising method for fabricating micro/nanofibers, which has shown great prospects in the biomedical fields such as 3D printing of porous scaffolds. The diameter of the melt electrospinning writing fiber can determine the resolution of the microstructure; thus, the controllability of the fiber diameter is of great significance to the whole fabrication process. In this paper, an orthogonal design experiment (six factors, three levels) was used to explore the impacts of six melt electrospinning parameters (melt temperature, collector speed, tip-to-collector distance, melt flow rate, voltage, and needle gauge) on the fiber diameter. In this experiment, the diameter of fibers obtained with the designed experimental parameters and conditions varied from 10.30 μm to 20.02 μm. The range analysis of orthogonal test results showed that the melt flow rate was the most important factor influencing the diameter of melt electrospinning writing fiber, while the voltage was the least influential factor. The variance analysis of orthogonal test results showed that melt temperature, collector velocity, tip-to-collector distance and melt flow rate had a significant influence on the diameter of melt electrospinning writing fiber. On the basis of the first-order regression equation, the fiber diameter of poly-ε-caprolactone can be accurately controlled, thus improving the engineering applications of poly-ε-caprolactone.

## 1. Introduction

Electrospinning is a method of generating micro/nanofibers from polymer liquid (solution or melt) under the action of a high-voltage electric field. Compared with the drafting method, template synthesis method, extrusion method, phase separation method, single-molecule self-assembly, and other methods for preparing nanofibers, electrostatic spinning technology provides a method for large-scale production of nanofibers [1,2]. Electrospinning uses electric field force rather than mechanical force to draft a polymer jet. The combination of electric field force and Coulomb force makes the polymer jet stretch to form nanofibers under a high electric field [3], and the resulting electrospinning nanofiber material (electrospinning film) has extremely high adsorption, filtration, separation, and resistance properties [4]. Electrospinning has been widely studied and applied in different fields such as composite materials, biocatalysis, personal protection, optical sensing, drug delivery, wound dressing, tissue culture, filtration and separation, sound absorption and noise prevention, and energy electronics [5,6].

However, electrospinning also has some disadvantages. One problem is that microfibers produced by conventional electrospinning are often randomly distributed. Researchers developed and improved the electrostatic spinning nozzle and collecting device to solve this problem. Dynamic mechanical devices have been used in electrospinning techniques, including drum fiber collectors [7], disc fiber collectors [8], fiber collectors with separate electrodes [9], and magnetic electrospinning [10]. However, the experimental platforms and techniques involved in these methods struggle to control fiber size and orientation. Although the ideal fiber size and orientation can be achieved by adjusting the processing parameters, the transverse direction of electrospinning films almost has no mechanical strength. Moreover, it is difficult to accurately control the fiber deposition site and the number of deposition layers; thus, the required 3D structure is not easy to obtain. Considering the problems of energy conservation and environmental protection, as well as precise deposition of nanofibers, researchers have gradually explored the low-voltage near-field electrostatic spinning technology. On the basis of traditional electrospinning, near-field direct writing electrospinning has gradually been developed by stabilizing the spinning jet in the initial stable stage.

Melt electrospinning writing (MEW) is a new technology of near-field direct writing electrospinning. Compared with traditional solution electrospinning, its solvent-free characteristics make it more biocompatible and applicable to a wider range of applications [11,12,13,14]. The utilization efficiency of polymer and the production efficiency of scaffold material can be greatly improved because there is no solvent volatilization [15]. Electrospinning molten mass has higher viscosity, higher yield, and lower electrical conductivity. In the spinning process, the influence of humidity and temperature on the spinning jet is lower. Therefore, the stable area of the spinning jet is longer, which greatly weakens the stirring effect and ensures the uniform and stable morphology of electrospinning fibers, thus accurately controlling the deposition of fibers [16]. On the basis of the above advantages, MEW has been widely evaluated by researchers and applied in tissue engineering [17], clinical medicine [18], micro/nano-processing [19], and other fields.

The great prospect of MEW technology has aroused people’s research. Dalton et al. [20] combined melt electrospinning equipment and a translation collecting plate. This enabled precise vertical stacking of the fiber layer by self-organizing. Tissue engineering mesh scaffolds with controllable structures and patterns can be designed and manufactured at multiple diameter scales ranging from 1 μm to 50 μm. You et al. [21] reduced the distance between the melt extrusion needle and the collecting plate from several centimeters to several millimeters for the first time, and the diameter of the melt electrospinning fiber was reduced from 20–50 μm to 1–2 μm at the minimum. The reduced deposition distance and the reduction of the applied electrostatic field led to a reduction in charge accumulation. This could further eliminate the end whipping effect of melt electrospinning, improve the precision of fiber deposition and the stability of the spinning process, and enhance the self-organizing effect of fibers. In their subsequent experiments, on the basis of the above theory, they constructed complex thin-walled structures with a diameter of electrospinning fibers <5 μm. At the same time, the number of fiber layers reached 200, the surface of the thin-walled structure was smooth, and there was no obvious thermal collapse. This study demonstrated the feasibility of transforming near-field melt electrospinning into a high-resolution but low-cost micro-3D structure-forming method.

With the progress of research, the effects of process parameters on the diameter of melt electrospinning fibers have been studied. Through orthogonal design experiments, He et al. [22] charted the variation trend of melt electrospinning fiber diameter with four melt electrospinning key process parameters: melt temperature, needle gauge, melt flow rate, and collector speed. After calculating the influence factors of each parameter, it was concluded that collector speed was the most influential factor for the diameter of the melt electrospinning fiber, while the needle gauge had the least significant influence on the diameter of the melt electrospinning fiber. Dayan et al. [23] designed experiments using the theories of design of experiment (DOE) and response surface methodology (RSM). The interaction of four key process parameters with regard to the diameter of the melt electrospinning fiber was studied: the collector speed, tip-to-nozzle distance, voltage, and the pressure applied to the syringe. Song et al. [24] simulated a changing electric field, and then studied the movement of the polymer chains, effect on jet diameter, and changes in the jet falling behavior at different control frequencies of the changing electric field. They found that, compared with the steady electric field, periodically changing the electric field could effectively improve the stretching of the molecular chain and reduce the jet diameter. A lower control frequency could accelerate the jet fall, thus obtaining finer fibers. Other researchers have also reported the influence of several important parameters on fiber diameter, including flow rate, applied voltage, and temperature [25,26,27]. However, we found that the previous studies did not comprehensively explain other parameters considered very important. We believe that a more comprehensive and clear comparison of processing parameters is needed, so as to have a clearer and intuitive cognition of the influence of process parameters on the diameter of melt electrospinning fibers.

In this paper, we conduct a qualitative analysis and quantitative prediction of the diameter of melt electrospinning fiber, which is the key unit of melt electrospinning micro-3D solid additive forming and the characterization unit of manufacturing resolution. On the basis of previous studies, we integrate many diameter influencing factors to intuitively observe the important role and influence of these factors. We use an orthogonal design method to verify the effects of the melt temperature, collector plate moving speed, tip-to-collector distance, melt flow rate, electrostatic field voltage, and needle aperture on the fiber diameter of the polymer. Our work is more comprehensive, and the conclusion of the correlation between the process parameters and fiber diameters provides some reference for subsequent research and production.

## 2. Experimental

### 2.1. Materials

In order to meet the needs of tissue engineering applications, a polymer with good biocompatibility is widely used in melt electrospinning. CAPA 6500 PCL (Poly-ε-caprolactone) pellets produced by Sweden Perstorp company (Malmö, Sweden) were used in experiment. PCL has gained huge interest as the polymer of choice for the construction of long-term implantable tissue engineering scaffolds due to its long degradation period, as well as superior rheological and viscoelastic properties [28]. It has good biodegradability, and it is beneficial for cell invasion and proliferation. Its relative molecular weight is 50,000 g/mol, with good characteristics of being transparent, nontoxic and tasteless in the melt state. Its low melting point of 58–60 °C and high thermal stability can constantly generate melt electrospinning fibers at low temperature without thermal decomposition.

### 2.2. Equipment

**Figure 1:**
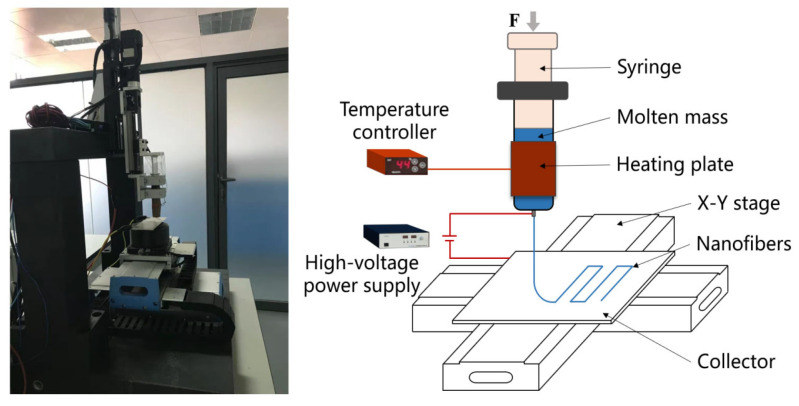
Self-designed melt electrospinning micro-3D forming system for the experiments.

The melt electrospinning systems built in various laboratories differ in terms of combination mode and performance index due to different research directions [12,29]. The experimental device we used is a self-designed computer numerical control 3D MEW printer, as shown in Figure 1. The critical device of the whole equipment was the precision syringe pump, on which the equipment was built. It consisted of a 10 mL luer lock glass syringe, a *Z*-axis linear motion module, and a closed-loop stepper motor. The syringe was wrapped with a heating plate, whose temperature was set by the temperature controller. An aluminum plate was mounted on a self-developed precision *X*–*Y* stage to serve as a collector. Two linear motors controlled the movement of the gantry platform in the *X*- and *Y*-directions. To achieve real-time location monitoring, the *X*–*Y* stage was equipped with limit switches and grating rulers. The path planning of the stage was realized by editing the multi-axis motion control card. The high-voltage DC power provided an external electrostatic field.

### 2.3. Electrospinning

The syringe was loaded with 19 G–23 G needles and was filled with PCL polymer. The heating process of the heating plates on the syringe was divided into two stages; the temperature in the first stage was set at 60 °C, while the second stage was set to the temperature required for the experiment. The *Z*-axis motion module was moved until the initial distance between the needle and the collector plate was 5 mm. The syringe was heated until the polymer melt temperature was uniform and stable; then, the melt injection pump was started, and the initial flow rate was set to 20 μL/h. The syringe needle was connected to the positive electrode of the high-voltage supply device, and the collector plate was connected to the negative electrode. Then, the high-voltage supply device was switched on and the initial output voltage was adjusted to 4.5 kV to make the Taylor cone emit a stable melt jet. After that, the *X*-axis and *Y*-axis were actuated to move along the designed path.

To eliminate the influence of uncontrollable environmental factors on the experiment, the PCL pellets were repeatedly heated and solidified at 60–80 °C for 2 h before each experiment. This measure eliminated bubbles caused by intergranular gaps in the melt. After each change in experimental parameters, the electrospinning fibers were deposited freely for 2 min to re-establish the stable melt electrospinning process. Then, electrospinning fibers were collected to eliminate the initial unstable disturbance.

To ensure the effectiveness of the fiber diameter, we randomly selected 10 sampling points for fiber diameter measurement and took the average value. Observation of the morphology and the stability of the deposited fibers was conducted through a precision optical microscope (MV5000 from Nanjing Jiangnan Novel Optics Co., Ltd., Nanjing, China) equipped with a Tucsen TCC-5.0 camera. Images of the deposited fibers were collected using the image acquisition software ISCapture 3.6.8, which is compatible with the Tucsen camera. Then, characterization of the fiber diameter was completed using the internal size measurement function of Image J 1.48.

## 3. Results and Discussions

The uniformity and stability of the melt electrospinning fiber are important to 3D forming quality. A uniform fiber diameter can avoid collapse and surface defects and ensure good structural strength and surface roughness [30,31]. Therefore, the establishment of an accurate prediction mechanism for the diameter of melt electrospinning fibers is the premise of 3D forming. This section analyzes the effect of six processing parameters on the diameter of the melt electrospinning fiber: melt temperature, collector speed, tip-to-collector distance, melt flow rate, voltage and needle gauge [32,33,34].

### 3.1. Construction of Orthogonal Test

Table 1 shows the six melt electrospinning processing parameters and their parameter ranges in the orthogonal experiment. The factor level was set to three, and the interval of factor parameters was evenly divided.

The orthogonal table can be expressed as *L_N_*(*q^s^*), where *N* represents the number of experiments, *s* represents the number of factors, and *q* represents the number of factor levels. An orthogonal table with *N* rows and *s* columns can be obtained. The construction of the orthogonal table follows the principle of uniform dispersion and neat comparability; that is, the occurrence frequency of every level of each factor is the same, and the collocation of any two levels of factors has the same frequency in the selected experiment. In order to conduct the subsequent analysis of variance of factor significance, errors in the test process were investigated, and a blank column was set at the end. The final form of the orthogonal table was a seven-factor three-level orthogonal test table *L_N_*(*3^7^*), which was designed by Minitab 18. A total of 27 experiments were conducted in accordance with the orthogonal design. The parameter distribution and the average fiber diameter of each experiment are shown in Table 2.

### 3.2. Effects of Processing Parameters on Fiber Diameter

It can be seen that, in this experiment, the MEW fibers with the minimum diameter (10.30 μm) were produced under the parameters of a temperature of 85 °C, a collector speed of 45 mm/s, a tip-to-collector distance of 10 mm, a melt flow rate of 20 μL/h, a voltage of 5.5 kV, and a needle gauge of 19 G. The fibers with the maximum diameter (20.02 μm) were produced under the parameters of a temperature of 95 °C, a collector speed of 25 mm/s, a tip-to-collector distance of 6 mm, a melt flow rate of 40 μL/h, a voltage of 4.5 kV, and a needle gauge of 21 G.

**Figure 2:**
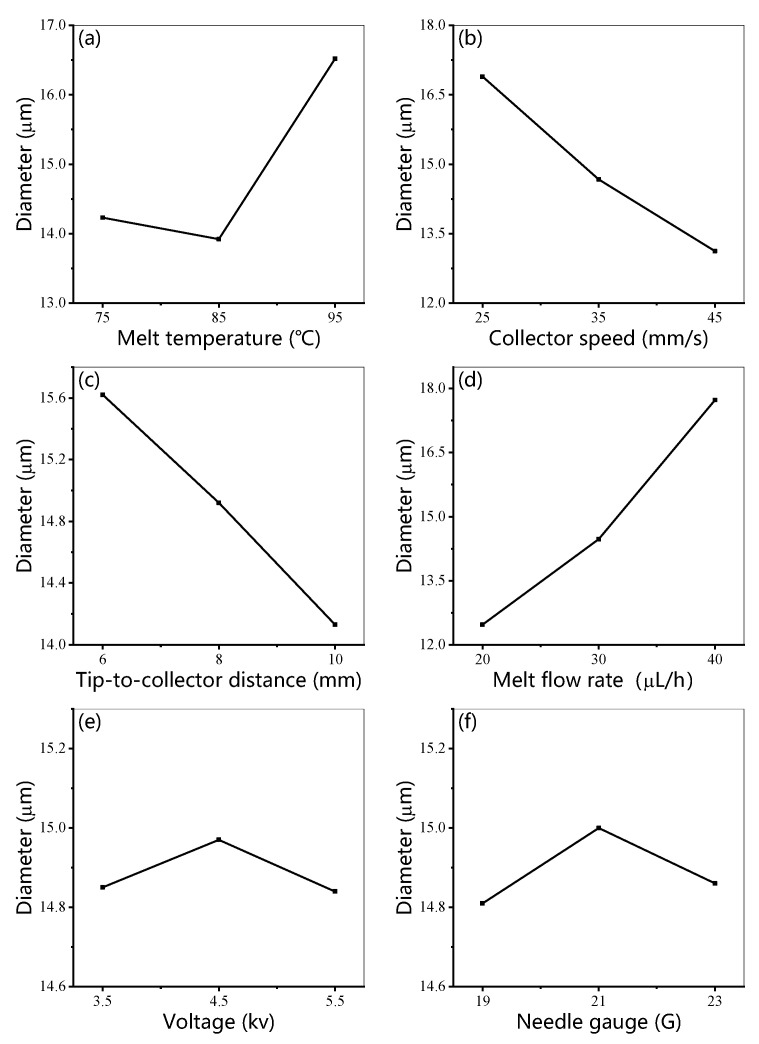
Diameter of melt electrospun fibers with respect to (**a**) melt temperature, (**b**) collector speed, (**c**) tip-to-collector distance, (**d**) melt rate, (**e**) voltage, and (**f**) needle gauge.

Figure 2 shows the variation trend of MEW fiber diameter with respect to the six processing parameters. This graph was plotted from the K values of the data in Table 2. At present, there is no reliable theoretical support for the effect of temperature on the diameter of fused electrospinning fibers. As illustrated in Table 2, we can see that, with the increase in melt temperature, the diameter of fibers decreased first and then increased. The influence of temperature on the diameter of melt electrospinning is mainly manifested in viscosity. In theory, the viscosity of molten PCL decreases with increasing temperature. In the case of low temperature, massive polymer chains move together, which leads to a higher viscosity. In the case of high temperature, the polymer chains can move freely, which results in a lower viscosity [35]. Under the action of the same electrostatic field, the charge on the surface of the low-viscosity (high-temperature) polymer easily overcomes the small surface tension to form the charged jet, and it is stretched into thinner fibers during the subsequent jet stretching process. However, when the melt temperature is too high, the melt viscosity is too low, resulting in a high fluidity of the melt. Therefore, more molten PCL flows out of the extrusion needle per unit time, which increases the diameter of the melt electrospinning fiber finally solidified on the collecting plate.

**Figure 3:**
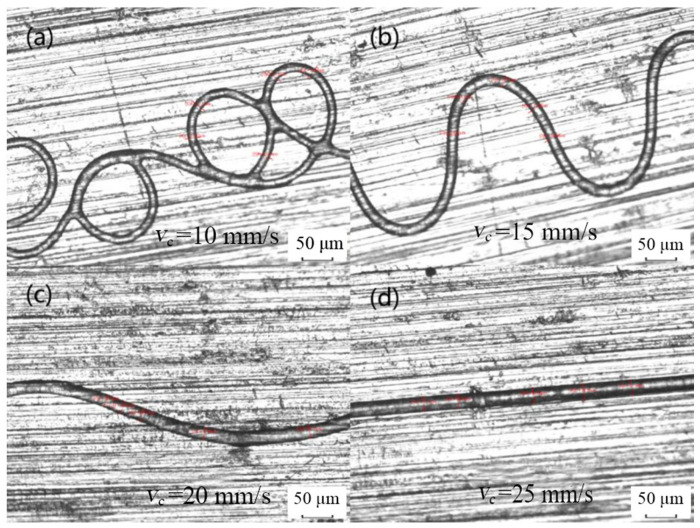
The morphology of the fibers changes with the increase in collector speed: (**a**) 10 mm/s; (**b**) 15 mm/s; (**c**) 20 mm/s; (**d**) 25 mm/s.

With the increase in moving speed of the collector, the diameter of the melt electrospinning fiber decreased continuously. The speed of the moving collector could directly affect the morphology of the fibers deposited on the substrate in the direct writing process, as shown in Figure 3. To obtain a straight uniform fiber in the direct writing process, a critical speed is always required for the moving collector to match the jet speed [20]. When this critical speed is exceeded, further increasing the speed of the collector would reduce the fiber diameter. This can be interpreted as the velocity difference producing extra drag force on the melt electrospinning fibers, which is similar to the uniform mechanical drawing process of the melt electrospinning fibers.

With the increase in tip-to-collector distance, the diameter of the melt electrospinning fiber decreased. Considering that, for a constant voltage, the electric field strength is generally higher when the collection distance is smaller, there is a larger electrostatic drawing effect on the fiber. If the collection distance is increased, there is another effect; the fiber has more time to cool down, which increases the viscosity and, therefore, the resistance of the jet to being stretched. As a result, the diameter of the fiber arriving at the collector is larger in these cases, although this outcome is not always observed [17].

With the increase in melt flow rate, the diameter of melt electrospinning fiber increased significantly. This result can be explained as follows: when the voltage is constant, the effect of increasing the material supplied to Taylor’s cone is to reduce the charge density in the material, thus reducing the electrostatic drawing effect on the jet and increasing the drag to be stretched, such that the jet is stretched less. Finally, the diameter of the fibers deposited on the substrate increases.

With the increase in applied high-voltage electrostatic field voltage, the diameter of the melt electrospinning fiber increased first and then decreased. However, in general, the change in diameter is not obvious. Previous studies have shown that a higher voltage results in a greater corresponding electrostatic force. The diameter of the fibers was reduced by increasing the voltage [36].

The diameter of the melt electrospinning fiber increased first and then decreased with the decrease in the needle gauge. This trend is consistent with previous reports [22]. This result can be interpreted as follows: when needles with larger inner diameters (smaller needle gauges) are used, charged jets tend to split during electrostatic spinning, resulting in reduced fiber diameters.

In conclusion, theoretically, in the melt electrospinning parameter space selected in this experiment, the melt electrospinning fiber with the minimum diameter can be obtained under the conditions of medium melt temperature, high collector speed, large tip-to-collector distance, low melt flow rate, high voltage, and small needle gauge.

### 3.3. Range Analysis

Range analysis was conducted on the orthogonal experiment data, and the results of range analysis were also listed in Table 2. The R-value (range) reflects the effect of each parameter on the average diameter. A larger R-value denotes a greater effect of the parameter on the fiber diameter. It can be seen that the R-value of each processing parameters (melt temperature, collector speed, tip-to-collector distance, melt rate, voltage, and needle gauge) was 2.61, 3.78, 1.50, 5.26, 0.13, and 0.18, respectively. According to the R-value, the importance of the effect of processing parameters on the diameter of melt electrospinning fibers can be listed in the following order: melt flow rate > collector speed > melt temperature > tip-to-collector distance > needle gauge > voltage. Melt flow rate appeared to have the greatest effect on fiber diameter, and it was significantly more important than the other parameters in terms of R-value. The voltage was the least important parameter, as the voltage levels designed in this paper had little effect on the fiber diameter.

### 3.4. Analysis of Variance (ANOVA)

Range analysis can only directionally describe the effect of different parameters on the diameter of MEW fibers. In order to confirm whether the influence of each parameter on the diameter of melt electrospinning fiber is statistically significant, ANOVA was conducted on the orthogonal experiment results, and the variance analysis results are shown in Table 3. The ANOVA of Table 2 was performed using the regression function of Statistical Product and Service Solutions (SPSS). The F-value indicates the influence (significance) of each experiment factor on the tested model. The associated *p*-value was used to assess F-value to indicate the statistical significance. It can be seen that *p*-values of melt temperature, collector speed, tip-to-collector distance, melt rate, voltage, and needle gauge were 0.000, 0.000, 0.000, 0.000, 0.370, and 0.165, respectively. According to statistical theory, factors with a *p*-value > 0.05 are insignificant factors; that is, their effect on the diameter of melt electrospinning fibers can be ignored. Therefore, it can be concluded that the factors with a significant effect on the diameter of the melt electrospinning fiber are melt temperature, collector speed, tip-to-collector distance, and melt rate.

### 3.5. Regression Analysis

The above work concluded the trends and significant parameters of fiber diameter with respect to individual parameters. Furthermore, MEW micro-3D structure formation requires the ability to obtain the diameter size of fibers according to the parameters. This requires a predictive model of MEW fiber diameter with respect to MEW parameters. Regression analysis was used to derive a regression equation describing the correlation between fiber diameter and six critical parameters. The function model is as follows: $y=b_{0}+\sum_{i=1}^{k}b_{i}x_{i}$. Regression coefficients *b_i_* reflect the weight of corresponding factors on the whole function.

Using the regress function of SPSS to solve the data in the Table 2, the results are shown in Table 4. According to the complex correlation coefficient, there is a strong correlation between the regression variables as the correlation coefficient approaches 1. Because of the F-statistic probability, which is related to the significant probability, the regression model is significantly effective. A large absolute value of the standardized indicates that its corresponding independent variable has a large effect on the dependent variable y. The results in the Table 4 correspond adequately to the previous analysis. The non-standardized results, on the other hand, were used to derive the regression equation for predictions of the dependent variable y, yielding a first-order regression equation:

(1) D=6.647+0.115T−0.189V−0.374H+0.263Q−0.006E+0.011G,

where *T* is the melt temperature, *V* is the collector speed, H is the tip-to-collector distance, *Q* is the melt flow rate, *E* is the voltage, and *G* is the needle gauge. According to the first-order regression equation, the fiber diameter of PCL can be accurately controlled. The high-precision printing of the PCL 3D scaffolds can benefit from our study. The good performance of the PCL gives it great potential for engineering applications. Our work can improve the applications of PCL including bone regeneration [37] and the construction of artificial periodontal ligament tissue [38], as well as cardiac and heart valves [39].

## 4. Conclusions

In the present paper, we fabricated straight single fibers using MEW. An orthogonal test was designed to verify the significant effect of six melt electrospinning process parameters on spinning fiber diameter. Four significant factors were screened out through the ANOVA. The results showed that the effect of six melt parameters on the diameter of melt electrospinning fibers can be listed in the following order: melt rate > collector speed > melt temperature > tip-to-collector distance > needle gauge > voltage. The factors with a significant impact on the output diameter of MEW were melt temperature, collector speed, tip-to-collector distance, and melt rate. The experimental results were consistent with the theoretical prediction. This experiment can provide an experimental reference for tissue engineering and other related industries. According to the first-order regression equation, the fiber diameter of PCL can be accurately controlled. High-precision printing of PCL 3D scaffolds will benefit from our study.

## Figures and Tables

**Table 1:** Orthogonal parameters and levels.

Level	Melt Temperature (°C)	Collector Speed (mm/s)	Tip-to-Collector Distance(mm)	Melt Flow Rate (μL/h)	Voltage (kv)	Needle GaugeG)
1	75	25	6	20	3.5	19
2	85	35	8	30	4.5	21
3	95	45	10	40	5.5	23

**Table 2:** Summary of experimental results and range analysis.

Number	Melt Temperature (°C)	Collector Speed (mm/s)	Tip-to-Collector Distance (mm)	Melt Flow Rate (μL/h)	Voltage (kv)	Needle Gauge (G)	Blank Column K	Diameter (μm)
1	75	25	6	20	3.5	19	1	14.35
2	75	25	6	20	4.5	21	2	14.69
3	75	25	6	20	5.5	23	3	14.59
4	75	35	8	30	3.5	19	1	13.48
5	75	35	8	30	4.5	21	2	13.78
6	75	35	8	30	5.5	23	3	13.59
7	75	45	10	40	3.5	19	1	14.41
8	75	45	10	40	4.5	21	2	14.72
9	75	45	10	40	5.5	23	3	14.47
10	85	25	8	40	3.5	21	3	19.00
11	85	25	8	40	4.5	23	1	18.77
12	85	25	8	40	5.5	19	2	18.60
13	85	35	10	20	3.5	21	3	10.66
14	85	35	10	20	4.5	23	1	10.57
15	85	35	10	20	5.5	19	2	10.30
16	85	45	6	30	3.5	21	3	12.51
17	85	45	6	30	4.5	23	1	12.44
18	85	45	6	30	5.5	19	2	12.39
19	95	25	10	30	3.5	23	2	17.40
20	95	25	10	30	4.5	19	3	17.11
21	95	25	10	30	5.5	21	1	17.49
22	95	35	6	40	3.5	23	2	19.81
23	95	35	6	40	4.5	19	3	19.79
24	95	35	6	40	5.5	21	1	20.02
25	95	45	8	20	3.5	23	2	12.07
26	95	45	8	20	4.5	19	3	12.89
27	95	45	8	20	5.5	21	1	12.14
$K_{1}$	14.23	16.89	15.62	12.47	14.85	14.81		
$K_{2}$	13.92	14.67	14.92	14.47	14.97	15.00		
$K_{3}$	16.52	13.12	14.13	17.73	14.84	14.86		
R	2.60	3.77	1.49	5.26	0.13	0.19		
Rank	3	2	4	1	6	5		

$K_{i}$ is the average diameter of melt electrospinning fibers at the same level of each parameter factor; i denotes their levels 1, 2, and 3; $R^{j}=maxK_{i}^{j}-minK_{i}^{j}$

**Table 3:** Summary of ANOVA results.

	DF	Adj SS	Adj MS	F-Value	*p*-Value
Melt temperature	2	35.528	18.2462	444.31	0.000
Collector speed	2	64.876	32.4379	789.11	0.000
Tip-to-collector distance	2	10.097	5.0487	122.82	0.000
Melt rate	2	126.749	63.3744	1541.70	0.000
Voltage	2	0.088	0.0439	1.07	0.370
Needle gauge	2	0.169	0.0844	2.05	0.165
Error	14	0.575	0.0411		
Total	26	239.83			

**Table 4:** Regression analysis results.

	Non-Standardized $b_{i}$	$\mathrm{Standardized}\;b_{i}$
$b_{0}$	6.647	
$b_{1}$	0.115	0.315
$b_{2}$	−0.189	−0.518
$b_{3}$	−0.374	−0.205
$b_{4}$	0.263	0.722
$b_{5}$	−0.006	−0.002
$b_{6}$	0.011	0.006
$r^{2}=0.930\;\;r=0.964\;\;p<0.001$		

## Data Availability

Not applicable.
